# Natural products in synthesis and biosynthesis II

**DOI:** 10.3762/bjoc.12.44

**Published:** 2016-03-03

**Authors:** Jeroen S Dickschat

**Affiliations:** 1 Kekulé-Institut für Organische Chemie und Biochemie, Rheinische Friedrich-Wilhelms-Universität Bonn, Gerhard-Domagk-Straße 1, D-53121 Bonn, Germany

**Keywords:** biosynthesis, natural products, synthesis

As a continuation of the first two Thematic Series in the *Beilstein Journal of Organic Chemistry* on natural products in 2011 and 2013 [1–2], it is my pleasure to present the third issue on this topic. Natural products continue to be an inspiring field of research and an important source of potent biologically active compounds. This was recently highlighted by the fact that last year’s Nobel Prize in Physiology and Medicine for the discovery of important drugs from natural sources that revolutionised the clinical treatment of parasite-borne infectious diseases was awarded to three natural product researchers: Youyou Tu (Academy of Chinese Medical Sciences) for the discovery of the terpenoid antimalaria drug artemisinin that is produced by the plant *Artemisia annua* [3], and to Satoshi Õmura and William C. Campbell for the discovery of avermectins isolated from the actinobacterium *Streptomyces avermitilis* at the famous Kitasato Institute and for the development of derivatives that are used as highly potent anthelmintics both for animal and human welfare [4]. Currently, also in the pharmaceutical industry, natural products are experiencing a revival as viable drug candidates, which is a pleasing development since many infectious diseases continue to threaten human health. From the numerous articles in daily newspapers, it is obvious that politicians have also realised the urgent need for new drugs and the potential associated with natural products and their derivatives. Certainly, the recent technological advances in many fields related to natural products (including synthetic methodology, as exemplified by a review article by Thomas Magauer and co-workers in this Thematic Series [5]), analytical chemistry [6], gene synthesis, and genome sequencing and editing [7] offer an efficient toolbox to natural products chemists. In addition, classical methods such as isotopic labelling experiments [8] continue to be important.

I thank all contributors that participated in this third Thematic Series on natural product chemistry in the *Beilstein Journal of Organic Chemistry* and also the whole team at the Beilstein-Institut for their professional work. I wish the readers of the present Thematic Series joyful reading and hopefully new inspiration for their own research.

Jeroen S. Dickschat

Bonn, February 2016
